# Sedation and Ventilator Weaning Bundle and Time to Extubation in Infants With Bronchiolitis: Secondary Analysis of the Sedation AND Weaning in Children (SANDWICH) Trial

**DOI:** 10.1097/PCC.0000000000003685

**Published:** 2025-01-23

**Authors:** Rebecca B. Mitting, Cliona McDowell, Bronagh Blackwood, Samiran Ray

**Affiliations:** 1 Paediatric Intensive Care Unit, Imperial College Healthcare NHS Trust, London, United Kingdom.; 2 Division of Anaesthetics, Pain Medicine and Intensive Care, Imperial College London, London, United Kingdom.; 3 Northern Ireland Clinical Trials Unit, the Wellcome-Wolfson Institute for Experimental Medicine, Queen’s University Belfast, Belfast, Ireland.; 4 Paediatric Intensive Care Unit, Great Ormond Street Hospital for Children NHS Foundation Trust and NIHR Biomedical Research Centre, London, United Kingdom.; 5 Respiratory, Critical Care and Anaesthesia Unit, Infection, Inflammation, and Immunity Division, University College London, London, United Kingdom.

**Keywords:** accidental extubation, bronchiolitis, failed extubation, sedation, ventilation, weaning

## Abstract

**OBJECTIVE:**

The Sedation and Weaning in Children (SANDWICH) trial of a sedation weaning and ventilator liberation bundle had a primary outcome of time to successful extubation, and showed significant but small difference. We explored the impact of the intervention on infants with bronchiolitis.

**DESIGN:**

Post hoc subgroup analysis of a cluster-randomized trial, 2018 to 2019 (ISRCTN16998143).

**PATIENTS:**

Surviving patients with bronchiolitis under 1 year of age in the SANDWICH trial (*n* = 784).

**INTERVENTIONS:**

Nil.

**MEASUREMENTS AND MAIN RESULTS:**

Time to successful extubation, and rates of unplanned and failed extubation were compared in patients exposed and not exposed to the intervention. To explore a site-level effect, we tested the correlation between the rate of unplanned and failed extubation at each trial site with the median time to successful extubation at that site. Of 784 patients (48%), 376 were exposed to the intervention. Median (interquartile range [IQR]) time to successful extubation was 69.6 (IQR 50.4–110.4) hours in patients exposed to the intervention and 86.4 (IQR 60–124.8) hours in non-exposed. Exposure to the SANDWICH intervention was associated with a 13% (95% CI, 1%–26%) reduction in time to extubation following adjustment for confounders. Thirty (3.8%) patients experienced unplanned extubation and 112 (14%) failed extubation. Patients who experienced failed extubation had an increased time to successful extubation, which remained significant after adjustment for confounders. At the site level, there was a negative correlation between failed extubation rate and median time to successful extubation (Spearman rho –0.53 [95% CI, –0.8 to –0.08], *p* = 0.02).

**CONCLUSIONS:**

In a secondary analysis of the SANDWICH trial, the subgroup of bronchiolitis patients showed that exposure to the intervention was associated with a clinically significant reduction in time to successful extubation. Although failed extubation was associated with increased duration of ventilation in an individual, sites with higher rates of failed extubation had a lower median duration of ventilation.

**RESEARCH IN CONTEXT**

- The Secondary Analysis of the Sedation and Weaning in Children (SANDWICH) trial of a sedation weaning and ventilator weaning protocol demonstrated a statistically significant but small improvement in time to extubation in patients exposed to the intervention.
- This small effect size may be owing to the heterogeneous population, including surgical and non-respiratory patients who may have had unrelated indications for continuing invasive ventilation.
- This report is a secondary, non-prespecified analysis of trial data, examining the effect size in infants with bronchiolitis.

**AT THE BEDSIDE**

- In the SANDWICH trial dataset, 2018 to 2019, we have identified 784 infants with bronchiolitis, 48% of whom were exposed to the trial intervention.
- Compared with the full trial results (ISRCTN16998143), the post hoc analysis shows that the trial intervention may have a more clinically significant impact on time to extubation in bronchiolitis patients receiving invasive mechanical ventilation.
- Pediatric Critical Care teams should consider the introduction of the SANDWICH bundle, as the reduction in time to successful extubation in infants with bronchiolitis may have an impact on seasonal bed pressures.

The Sedation and Weaning in Children (SANDWICH) trial was a stepped-wedge cluster-randomized trial of a sedation and ventilator liberation protocol carried out from 2018 to 2019 (ISRCTN16998143) (1). The intervention group had a median time to successful extubation of 64.8 hours and the control group 66.2 hours. Although a statistically significant result, there remains some uncertainty around the clinical significance of the difference. Implementation of the bundle within U.K. PICUs has so far been limited (2). The effect size of the intervention may, however, have been impacted by non-modifiable patient factors such as the requirement for repeated surgical intervention or airway protection.

Bronchiolitis represents a relatively homogenous subgroup of patients intubated for respiratory failure, forming a large proportion of patients requiring invasive mechanical ventilation (IMV) in PICU. The diagnosis is also important in terms of resource management, as its seasonal occurrence creates pressure on PICU beds during the winter season (3–6). Our group has previously investigated factors associated with variation in duration of IMV in infants with acute viral bronchiolitis, defined as having presented with cough, tachypnoea chest recession, or apnea without other clinical signs within the first year of life (1, 7). Unplanned and failed extubations are both adverse events associated with harm in children and may be affected by a protocolized weaning intervention (8–10). The frequency of unplanned extubation was higher in the SANDWICH trial in children who were exposed to the intervention than those who were not, although there was no difference in the extubation failure rate. This finding highlights the trade-off at a population level between optimal achievement of sedation and ventilation weaning with the aim of early extubation, and the potential to increase failed and unplanned extubation events. Many unplanned extubation episodes do not require reintubation (11), suggesting that clinicians may be exercising over-cautious approaches to extubation, to avoid extubation failure owing to its association with poor outcomes (9, 10).

Therefore, we aimed to examine the effect of the SANDWICH intervention on time to successful extubation in a group of infants with bronchiolitis. In addition, we also explored: 1) the effect of the SANDWICH intervention on the frequency of unplanned and failed extubation in infants with bronchiolitis, 2) the impact of both on-time to successful extubation in the context of the trial intervention, and 3) the relationship between frequency of failed and unplanned extubation at each trial site and the median time to successful extubation at that site. Our primary hypothesis is that the effect size of the intervention in the bronchiolitis subgroup is larger than that observed in the full trial, and therefore clinically significant. Second, although unplanned and failed extubation has a detrimental effect at the patient level, at the site level having a very low frequency of unplanned and failed extubation may be associated with a longer than average duration of IMV.

## MATERIALS AND METHODS

In this post hoc analysis of the SANDWICH trial dataset, we included surviving patients with a primary diagnostic code of bronchiolitis and age less than 12 months at the time of trial entry. The sub-population was defined before the post hoc analysis. The SANDWICH protocol and results have previously been published (1, 12). The clinical trial registration was isrctn.org Identifier, ISRCTN16998143, and the National East Midlands research ethics committee approved the protocol (17/EM/0301) on September 12, 2017. Procedures were followed in accordance with the ethical standards of the responsible committee (National East Midlands Research Ethics Committee) and with the Helsinki Declaration of 1975.

An opt-out consent approach was used with the distribution of study leaflets to parents. There was no requirement for written or oral informed consent. The Northern Ireland Clinical Trials Unit managed the trial. Data collection was managed through the mandatory national registry Pediatric Intensive Care and Audit Network) of PICU admissions with additional items recorded on electronic case report forms. Independent oversight was provided through the steering and data and safety monitoring committees convened by the U.K. National Institute of Health Research. Results are reported according to the Strengthening the Reporting of Observational Studies in Epidemiology statement on reporting observational cohort studies (13).

### Data Collection

Data were extracted from the following fields in the case report form: gestational age at birth, age at admission, positive end-expiratory pressure (PEEP), positive inspiratory pressure (PIP), Fio_2_ at 08:00 hours on the first day following entry into the study, time to successful extubation in hours, duration of PICU stay in days, Pediatric Index of Mortality 3 score (14), use of continuous IV sedation on days 1 and 2 of admission, the frequency of unplanned extubation (defined as dislodgement of the ETT from the trachea, without the intention to extubate immediately and without the presence of airway competent clinical staff in the bed space appropriately prepared for the procedure) (15), and emergency reintubation for failed extubation (defined as a requirement for emergency reintubation within 48 h of an attempt to separate from IMV) (16). Where Fio_2_ was missing, Fio_2_ was imputed to the median value for the rest of the cohort.

Infants were considered as term if gestational age was not entered, and corrected gestation age (CGA) was calculated from gestational age at birth and age at admission to study. Infants were categorized into three categories of prematurity: less than 32 weeks gestation at birth, 32–37 weeks gestation at birth, and greater than 37 weeks gestation at birth. Children were excluded if they did not survive PICU discharge.

### Statistical Analyses

Univariable analysis was used to compare time to successful extubation with and without exposure variables, using the Mann-Whitney *U* test. Correlation between continuous variables was assessed using Spearman rank. The frequency of events for infants exposed and not exposed to the intervention was compared using the chi-square test.

Generalized linear models using a gamma distribution with a log-link were used to assess the differences in time to successful extubation with and without exposure to the intervention, and with and without unplanned and failed extubation, after adjustment for known confounders with the trial site as a random effects variable. The site was a random effects variable because one site crossed over from usual care to intervention delivery every four weeks, meaning the proportion of patients exposed to intervention was different at each site. The same analysis was repeated with only children who experienced failed extubation included, to explore the specific impact of the intervention in this subgroup. This subgroup was selectively analyzed as both the intervention, failed extubation, and time to successful ventilation are likely interdependent—a combined model including the intervention and failed extubation would lead to collider bias.

Previous work by our group and others showed an association between baseline Oxygen Saturation Index (OSI) and the duration of IMV in bronchiolitis (2, 7, 17). OSI data were not available from the SANDWICH dataset, therefore, we used Fio_2_ as a surrogate in our primary model owing to data completeness. To explore the impact of the inclusion of IMV parameters within the model, we combined the available PIP, PEEP, and Fio_2_ values with median imputation to calculate OSI assuming an Inspiration to Expiration (I:E) ratio of 1:2 and pulse oximetry oxygen saturations (Spo_2_) of 97% and repeated the regression analysis (18, 19).

Analyses were performed using R (20), dplyr package (Vienna, Austria) was used for data manipulation, and lme4 for linear regression (21, 22). A *p* value of less than 0.05 was considered statistically significant. No correction was made for multiple comparisons in the univariable analysis.

## RESULTS

**Figure 1.**
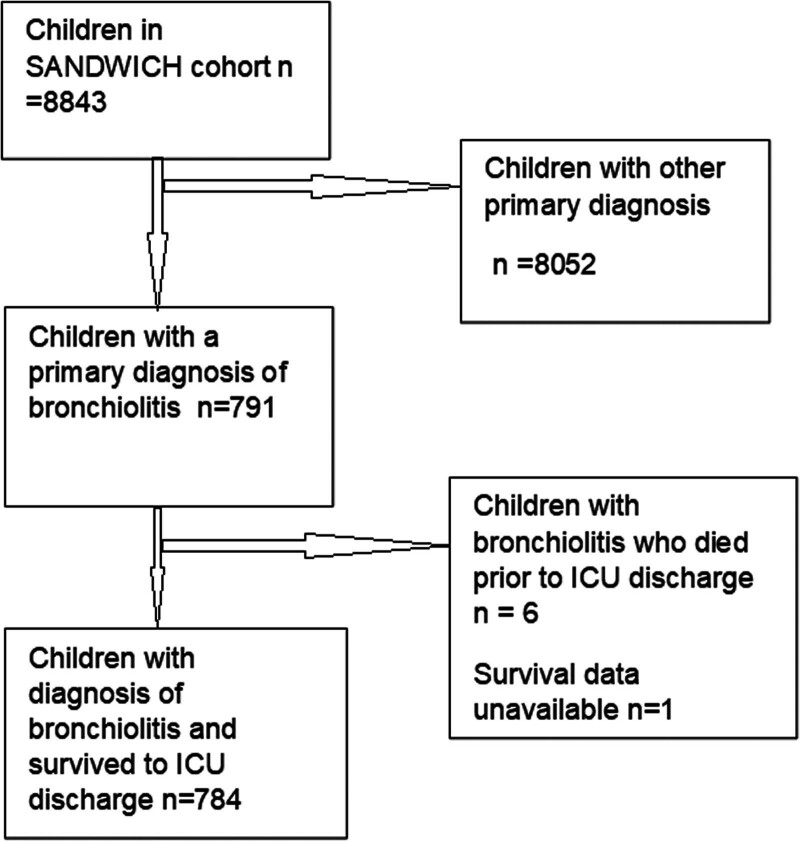
Consolidated Standards of Reporting Trials diagram for inclusion into the post hoc analysis. SANDWICH = Secondary Analysis of the Sedation and Weaning in Children.

Seven hundred ninety-one children from the SANDWICH dataset had a diagnosis of bronchiolitis and were younger than 1 year old at admission to the study. All but 1 of these children were expected to receive IMV for greater than 24 hours as categorized at the time of enrollment to SANDWICH. Six children did not survive PICU discharge (0.76%) and data on survival were unavailable for one patient. This left 784 patients for analysis (**Fig. 1**). These patients were cared for in 17 trial sites, 2 of which had small numbers (2 and 5 patients): these were included in the per-patient analysis but excluded for site-level analysis. Fio_2_ was available for all but five patients.

Baseline characteristics were similar between infants who were and were not exposed to the intervention (**Table 1**). Using univariable analysis, the median (interquartile range [IQR]) time to successful extubation in SANDWICH intervention exposed vs. non-exposed infants was 69.6 (IQR 50.4–110.4) vs. 86.4 (IQR 60–124.8) hours, *p* < 0.001 (Mann-Whitney *U* test). PICU stay in exposed vs. non-exposed infants was a median of 5 (IQR 4–7.25) vs. 6 (IQR 4–8) days, *p* = 0.05. There was no difference in the frequency of unplanned or failed extubation between exposed and non-exposed infants, likely owing to the relatively small numbers in each group (Table 1).

**TABLE 1.** Univariable Analysis–Baseline Characteristics and Outcomes of the Cohort, and in Patients Who Were and Were Not Exposed to the Sedation and Weaning in Children Intervention

Variable	Entire Cohort, *n* = 784	Exposed to Intervention, *n* = 383 (49%)	Not exposed to Intervention, *n* = 401 (51%)	*p*
Corrected gestational age in weeks relating to 40 wk, median (IQR)	4.3 (–1, 19)	4.3 (–0.8 to 21.7)	4.3 (0.7, 17.3)	0.64
Fraction of inspired oxygen	0.35 (0.3, 0.45)	0.35 (0.3, 0.45)	0.35 (0.30, 0.48)	0.97
Gestation (completed weeks), *n* (%)				
Term	454 (57.9)	223 (58.2)	231 (57.6)	
32–37	185 (23.5)	88 (23.0)	97 (24.2)	
< 32	145 (18.5)	72 (19.0)	73 (18.2)	0.96
Time to successful extubation (h), median (IQR)	79.2 (55.2, 115.2)	69.6 (50.4, 110.4)	86.4 (60, 124.8)	**< 0.001**
Days in PICU, median (IQR)	6 (4–8)	5 (4, 7.25)	6 (4, 8)	0.05
Frequency of unplanned extubation, *n* (%)	30 (3.8)	17 (4.4)	13 (3.2)	0.46
Frequency of failed extubation, *n* (%)	112 (14)	49 (13)	63 (15.7)	0.34

IQR = interquartile range.

Chi-square tests were used to compare categorical variables and Mann-Whitney *U* tests were used to compare continuous variables. *p* < 0.05 was considered significant and in boldface font.

After adjusting for covariates in the generalized linear model (prematurity, CGA, Fio_2_), exposure to the SANDWICH intervention was associated with a 13% reduction in the geometric mean time to successful extubation (**Table 2**).

**TABLE 2.** Generalized Linear Model

Variable	Coefficient (Expressed as Exponential of Coefficient as Log-Linked Model)	95% CI	*p*
Exposure to intervention	0.87	0.74, 0.999	**0.03**
Fraction of inspired oxygen	2.34	2.2, 2.5	**< 0.001**
Corrected gestational age (wk)	0.997	0.994, 1	0.09
Term gestation	Reference		
Prematurity 32–37 wk	1.1	0.95, 1.12	**0.12**
Prematurity < 32 wk	1.56	1.24, 1.9	**< 0.001**

Gamma distribution with a log link as the duration of IMV is left-skewed) demonstrating association between exposure to Sedation and Weaning in Children trial intervention and time to successful extubation following adjustment for known confounders. Shown is the exponential of the coefficients with 95% CIs, and *p* < 0.05 were considered significant and in boldface font.

We explored the effect of unplanned and failed extubation on the time to successful extubation separately. **Tables S1** and **S2** (http://links.lww.com/PCC/C586) show the baseline characteristics and outcomes in children who did and did not experience unplanned and failed extubation. Experience of unplanned extubation was not associated with a difference in time to successful extubation following adjustment for confounders. After adjusting for confounders, the experience of failed extubation was associated with a 124% increase in geometric mean time to successful extubation (**Tables S3** and **S4**, http://links.lww.com/PCC/C586).

The SANDWICH intervention could potentially affect both the decision to extubate and the outcome. Data are not available within the trial dataset to explore the timing of failed extubation to examine whether failed extubation followed a period of prolonged ventilation, or failed extubation occurred early, leading to a longer period of ventilation afterward. To explore this further, we considered the effect of the intervention in only patients who experienced extubation failure (*n* = 112). The median time to successful extubation was 119.6 (IQR 85.8–176.3) hours in children exposed to the intervention and 152.6 (IQR 99.3–265.9) in those who were not. On univariable analysis, this comparison was not significantly different (**Table S5**, http://links.lww.com/PCC/C586). Following multivariable analysis, however, there was an association between exposure to the SANDWICH intervention and a reduction in time to successful extubation (**Table 3**).

**TABLE 3.** Generalized Linear Model

Variable	Coefficient (Expressed as Exponential of Coefficient as Log-Linked Model)	95% CI	*p*
Exposure to intervention	0.71	0.41–1	**0.03**
Fraction of inspired oxygen	1.1	0.27–1.93	0.8
Corrected gestational age (wk)	1.0	0.99–1.01	0.93
Term gestation	Reference		
Prematurity 32–37 wk	0.93	0.64–1.22	0.66
Prematurity < 32 wk	1.28	0.97–1.59	0.11

Gamma distribution with a log link as the time to successful extubation is left-skewed) demonstrating the association between exposure to Sedation and Weaning in Children trial intervention and time to successful extubation following adjustment for known confounders including only patients who experienced a failed extubation (*n* = 112). Shown is the exponential of the coefficients with 95% CIs, and *p* < 0.05 were considered significant and in boldface font.

**Figure 2.**
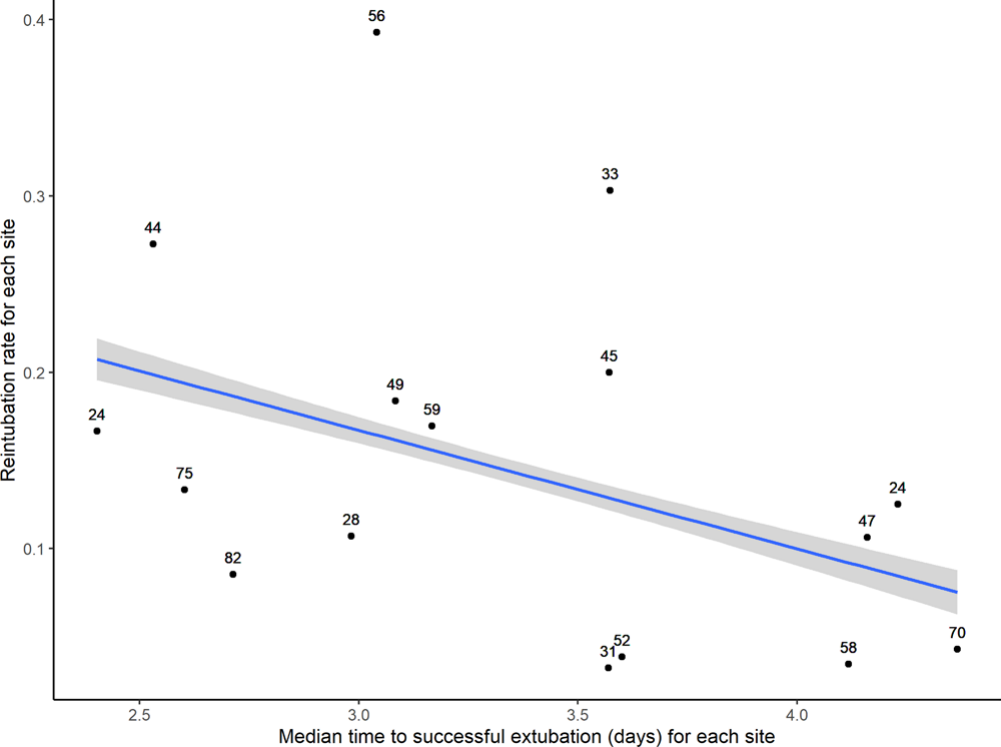
Frequency of reintubation at a site in comparison with median time to extubation at that site. The number next to the point reflects the volume of cases at the site (sites with < 5 patients not included).

**Figure 2** demonstrates the relationship between the frequency of failed extubation by site and the median time to successful extubation at that site. This correlation has a Spearman rank rho of –0.53 (95% CI, –0.8 to –0.08; *p* = 0.02) demonstrating a negative correlation between reintubation rate at a site and median time to extubation. There was no correlation between the frequency of unplanned extubation at a site and the time to extubation.

**Table S6** (http://links.lww.com/PCC/C586) shows the distribution of variables and duration of invasive IMV by site of study. The frequency of failed extubation at a site ranged from none to 39% and the frequency of unplanned extubation ranged from none to 10% of patients.

The multivariable models used adjustment for Fio_2_ as a marker of severity of lung disease. Given that the association between OSI and duration of IMV in bronchiolitis has previously been described, we imputed the OSI values for individual patients using PIP, PEEP, and Fio_2_. PIP and PEEP were available for 722 of 784 patients; for the remaining 62 patients, these were imputed to the median value for the cohort. **Table S7** (http://links.lww.com/PCC/C586) shows the generalized linear model coefficients with adjustment for imputed OSI instead of Fio_2_. The association between exposure to the SANDWICH intervention and a reduction in time to successful extubation remains.

## DISCUSSION

In this post hoc analysis of the 2018 to 2019 SANDWICH randomized trial dataset, we have studied a large subgroup of bronchiolitis patients and demonstrated that exposure to the trial intervention was associated with a 17-hour reduction in time to successful extubation. Although unplanned extubation was not associated with a difference in the time to extubation, failed extubation was associated with a significant increase. However, at the site level, a higher rate of failed extubation correlated with a lower median time to successful extubation.

In the SANDWICH trial, the median time to successful extubation was 64.8 hours in the intervention-exposed group and 66.2 in the controls. In the subgroup of infants with bronchiolitis, we have found that the median times were 69.6 hours and 86.4 hours, respectively; that is a difference of 17 hours in comparison to 2 hours in the whole trial population. This difference translates into a 1-day decrease in PICU length of stay, which was not seen in the full trial dataset. This decrease in resource utilization could be important in persuading clinicians to implement the intervention. The seasonal nature of bronchiolitis, occurring in winter epidemics is of particular importance to PICU clinicians, as it places a significant strain on resources worldwide (3–6). Facilitation of early discharge and patient flow have been highlighted as key strategies to maintain safe levels of PICU bed occupancy during winter peaks (4). The impact on cost-saving is also important as critical care costs are a major driver of the overall increase in healthcare costs related to bronchiolitis over the past 20 years (23).

Unplanned extubation occurred in 4.4% of children who were exposed to the intervention and 3.2 % of children who were not exposed. Following adjustment for confounders unplanned extubation was not associated with increased time to successful extubation. Although not significantly different in this subgroup of children with bronchiolitis, 11 of 30 (37% [95% CI, 19%–54%]) children who experienced unplanned extubation in the cohort were not re-intubated. This observation is consistent with other PICU studies (11). There will be valid clinician concerns regarding the introduction of an intervention that may increase the frequency of unplanned extubation, as seen in the full SANDWICH trial dataset. The lack of a significant difference seen in this bronchiolitis subgroup may be a result of a lack of statistical power. Nevertheless, over a third of the children who experienced unplanned extubation were not re-intubated, suggesting that there is potential to further shorten IMV duration in some cases. The challenge would be to do so safely and under controlled circumstances with more robust methods of recognizing extubation readiness.

Failed extubation has been associated with increased total duration of IMV, increased length of stay, as well as mortality in both adults and children (9, 10). The frequency of failed extubation in the current cohort was 14%, which is similar (perhaps slightly higher) to other PICU populations with a reported frequency between 6 and 10% (9, 24, 25), slightly higher than the SANDWICH trial population as a whole (1). In postoperative cardiac patients, extubation failure frequency has been described between 5% and 8% (10, 26). In the setting of U.K. PICU randomized controlled trials, frequency has been 11.5%–13.3% (1, 27). Specifically in bronchiolitis patients, three cohort studies have shown rates of 6%–9% (2, 7, 28). Patients who experienced failed extubation were ventilated for a median of 137 hours, compared with 72 hours for those who did not; and, failed extubation was associated with a large increase in time to successful extubation following adjustment for known confounders—which is consistent with previous studies (9, 10).

The SANDWICH intervention, however, was still associated with a shorter time to successful extubation in patients who did experience a failed extubation. A ventilator and sedation weaning bundle which includes extubation readiness testing is designed to impact a clinician’s decision to extubate and therefore could be causally linked to extubation failure. That the intervention retained a benefit in those patients who experienced a failed attempt at extubation, suggests that the intervention is unlikely to adversely affect this high-risk group.

As this work has identified heterogeneity of treatment effect within the SANDWICH trial cohort, it necessarily raises the possibility that a different subgroup did not benefit from the intervention or even experienced harm. This may be an area for future work.

At a site level, there was a negative correlation between the frequency of failed extubation at a site and the median time to successful extubation at that site. Avoidance of failed extubation is beneficial at a patient level. This is traded against potentially over-cautious attitudes to extubation, which may increase the average duration of IMV at a population level. The negative correlation between the rate of failed extubation and the duration of ventilation at the site level may represent this. Future work in this area should therefore include the use of data from large cohorts of patients to gain a better understanding of the epidemiology of failed extubation to develop personalized medicine and improve our ability to predict extubation success. This could help us to predict, not only when to extubate our patients, but which patients to extubate with the support of post-extubation noninvasive ventilation.

### Limitations

There are several limitations of the current post hoc analysis of the SANDWICH study. First, although it is a subgroup analysis of a trial dataset and therefore underpowered to demonstrate differences in the trial intervention, the subgroup is a comparably large one of children with bronchiolitis. This does allow us to draw some conclusions and generate hypotheses. Data for some confounding variables (e.g., presence or absence of comorbidity, presence or absence of bacterial coinfection) were not available within the SANDWICH dataset. Bronchiolitis was selected as a diagnosis for this analysis, as it is a common condition, with a relatively homogenous population in terms of diagnosis and age. However, it is likely that the group is not entirely homogenous, and it is possible that there are differences in severity of illness or frequency of comorbidity not adjusted for in this analysis. Second, data for Fio_2_ were missing for five patients, and we had to impute values for the calculation of OSI, to be consistent with previous analyses. This may have led to inaccuracies in OSI assessment. Third, this analysis is exploratory, and therefore causation cannot be demonstrated. It is not possible to state whether failed extubation caused prolonged ventilation, or that infants who underwent prolonged ventilation for other reasons experienced higher rates of reintubation. Fourth, the exploratory finding of a correlation between higher rates of reintubation and lower median duration of ventilation should not be interpreted at this stage as a causal relationship, as it is a finding that does not consider other factors such as case mix. Fifth, a limitation of the analysis by the site is that SANDWICH sites will have transitioned to the intervention at different time points, so the proportion of patients exposed to the intervention is not evenly distributed (Table S6, http://links.lww.com/PCC/C586). Sixth, the number of patients per site was too small to examine the impact of the intervention on the rate of failed and unplanned extubation at a site level. For example, the site-level correlation may have been influenced by a small number of sites with very high failed extubation rates.

## CONCLUSIONS

This subgroup analysis of the 2018 to 2019 SANDWICH trial suggests that the effect size of the intervention bundle is larger when considering a cohort of patients with bronchiolitis as the admitting diagnosis. This observation may be a more accurate estimate of the intervention effect and is significant when considering resource management during seasonal respiratory virus peaks. Furthermore, failed extubation is associated with prolonged IMV in infants with bronchiolitis, and future work should aim to improve our ability to predict occurrence, to minimize risk for the individual, without prolonging IMV duration for the population.

## Supplementary Material
