# Supplementary material for: Sedation and Ventilator Weaning Bundle and Time to Extubation in Infants With Bronchiolitis: Secondary Analysis of the Sedation AND Weaning in Children (SANDWICH) Trial
Source: Pediatr Crit Care Med. 2025 Jan 23;26(4):e423–31. doi: 10.1097/PCC.0000000000003685 (PMC11960679; doi:10.1097/PCC.0000000000003685)
Supplement: Supplementary file 1 [file pcc-26-e423-s001.docx]

Supplemental Digital Content for:

Sedation and ventilator weaning bundle and time to extubation in infants with bronchiolitis: secondary analysis of the Sedation AND Weaning In CHildren (SANDWICH) trial

Contents

1. Table S1 – Uni-variable analysis - baseline characteristics and outcomes of the cohort, and in patients who did and did not experience unplanned extubation (Page 2).
2. Table S2 – Uni-variable analysis - baseline characteristics and outcomes in patients who did and did not experience failed extubation (Page 3).
3. Table S3 – Generalised linear model (gamma distribution with a log link as time to successful extubation is left skewed) demonstrating associations between experience of unplanned extubation and time to successful extubation following adjustment for known confounders. (Page 4)
4. Table S4 – Generalised linear model (gamma distribution with a log link as time to successful extubation is left skewed) demonstrating associations between failed extubation and time to successful extubation following adjustment for known confounders. (Page 5)
5. Table S5 – Uni-variable analysis - baseline characteristics and outcomes of the cohort, and in patients who were and were not exposed to the SANDWICH intervention, only including patients who experienced failed extubation (n =112). (Page 6)
6. Table S6 - the distribution of variables and median time to successful extubation by site of study. (Page 7)
7. Table S7– Generalised linear model (gamma distribution with a log link as duration of IMV is left skewed) demonstrating associations between exposure to the SANDWICH intervention and time to successful extubation following adjustment for known confounders with OSI replacing FiO2 to explore impact of inclusion of pressure within the model. (Page 8)

| **Variable** | **Patients who experienced unplanned extubation**  **n = 30 (3.8%)** | **Patients who did not experience unplanned extubation**  **n = 754 (96%)** | **P value (Mann-Whitney U Test for continuous variables chi-squared test for categorical)** |
| --- | --- | --- | --- |
| PIM3 score median (IQR) | 0.007  (0.005,0.008) | 0.006  (0.004,0.007) | 0.22 |
| CGA (weeks in relation to 40 weeks)  median (IQR) | 0.7  (-1.2,3.2) | 4.3  (-0.7,19) | 0.07 |
| FiO_2_  median (IQR) | 0.38  (0.31,0.44) | 0.4  (0.3,0.45) | 0.35 |
| Gestation at birth  (completed weeks), n (%)  Term  32-37  <32 | 15 (50)  7 (23)  8 (27) | 439 (58)  178 (24)  137 (18) | **0.48** |
| Time to successful extubation in hours median (IQR) | 108.2  (65.1,149.9) | 77.9  (54.4,115.7) | 0.05 |
| Days in PICU  median (IQR) | 6.5  (5,8) | 6  (4,8) | 0.19 |

**Table S1 – Uni-variable analysis:** Baseline characteristics and outcomes of the cohort, and in patients who did and did not experience unplanned extubation. Chi-squared tests were used to compare categorical variables, Mann-Whitney U tests were used to compare continuous variables. A p value <0.05 was considered significant. No adjustment for multiple comparisons.

| **Variable** | **Patients who experienced failed extubation**  **n = 112 (14%)** | **Patients who did not experience failed extubation**  **n = 672 (86%)** | **P value (Mann-Whitney U Test for continuous variables chi-squared test for categorical)** |
| --- | --- | --- | --- |
| PIM3 score median (IQR) | 0.006  (0.004,0.008) | 0.006  (0.004,0.007) | 0.37 |
| CGA (weeks in relation to 40 weeks)  median (IQR) | 2.8  (-1.7,13) | 4.3  (-0.7,2.1) | 0.11 |
| FiO_2_ | 0.35  (0.3,0.5) | 0.35  (0.3,0.45) | 0.9 |
| Gestation at birth  (completed weeks), n (%)  Term  32-37  <32 | 47 (42)  34 (30)  31 (28) | 407 (61)  151(22)  114 (17) | **<0.001** |
| Time to successful extubation (hours)  median (IQR) | 136.8  (91.2,228) | 72  (50.4,108) | **<0.001** |
| Days in PICU median (IQR) | 8  (6,13) | 5  (4,7) | **<0.001** |

**Table S2 – Univariable analysis:** Baseline characteristics and outcomes in patients who did and did not experience failed extubation. Chi-squared tests were used to compare categorical variables, Mann-Whitney U tests were used to compare continuous variables. A p value <0.05 was considered significant. No adjustment for multiple comparisons.

| **Variable** | **Coefficient (expressed as exponential of coefficient as log-linked model)** | **95%CI** | **p value** |
| --- | --- | --- | --- |
| Unplanned extubation | 1.1 | 0.86,1.34 | 0.4 |
| FiO_2_ | 2.3 | 1.98,2.62 | **<0.001** |
| Corrected gestational age (weeks) | 0.997 | 0.994,1 | 0.12 |
| Term gestation | Ref |  |  |
| Prematurity 32-37 weeks | 1.09 | 0.97,1.21 | 0.14 |
| Prematurity <32 weeks | 1.56 | 1.43,1.69 | **<0.001** |

**Table S3 – Generalised linear model:** Gamma distribution with a log link as time to successful extubation is left skewed demonstrating associations between experience of unplanned extubation and time to successful extubation following adjustment for known confounders.

| **Variable** | **Coefficient (expressed as exponential of coefficient as log-linked model)** | **95%CI** | **p value** |
| --- | --- | --- | --- |
| Failed extubation | 2.24 | 2.12,2.36 | **<0.001** |
| FiO_2_ | 2.47 | 2.2,2.8 | **<0.001** |
| Corrected gestational age (weeks) | 0.99 | 0.99,1 | 0.1 |
| Term gestation | Ref |  |  |
| Prematurity 32-37 weeks | 1.04 | 0.94,1.1 | 0.4 |
| Prematurity <32 weeks | 1.42 | 1.3,1.5 | **<0.001** |

**Table S4 – Generalised linear model:** Gamma distribution with a log link as time to successful extubation is left skewed) demonstrating associations between failed extubation and time to successful extubation following adjustment for known confounders.

| **Variable** | **Exposed to Intervention**  **n = 48 (44%)** | **Not exposed to Intervention**  **n = 63 ( 56%)** | **P value for univariable analaysis (Chi- Squared for categorical variables, Mann- Whitney U for continuous variables)** |
| --- | --- | --- | --- |
| Corrected gestational age  (weeks in relation to 40 weeks)  median (IQR) | 2.7  (-1.3-21.7) | 3  (-1.7,10) | 0.26 |
| FiO_2_  median (IQR) | 0.4  (0.3,0.5) | 0.35  (0.3,0.44) | 0.12 |
| Gestation at birth  (completed weeks), n (%)  Term  32-37  <32 | 19 (40)  14 (29)  15 (31) | 27 (43)  20 (32)  16 (25) | 0.82 |
| Time to successful extubation in hours median (IQR) | 119.6  (85.8,176.3) | 152.6  (99.3,265.9) | 0.08 |
| Days in PICU  median (IQR) | 7  (6,11) | 9  (6.5,17) | **0.04** |

**Table S5 – Univariable analysis:** Baseline characteristics and outcomes of the cohort, and in patients who were and were not exposed to the SANDWICH intervention, only including patients who experienced failed extubation (n =112). Chi-squared tests were used to compare categorical variables, Mann-Whitney U tests were used to compare continuous variables. A p value <0.05 was considered significant. No adjustment for multiple comparisons.

| **Site** | **Number of patients from site** | **Proportion of patients with failed extubation** | **Proportion of patients with failed extubation -not exposed** | **Proportion of patients with failed extubation – exposed to intervention** | **Proportion of Patients with Unplanned Extubation** | **Fraction of inspired oxygen median(IQR)** | **Proportion of Patients at site exposed to intervention** | **Proportion of patients born < 32 weeks gestation** | **Median Time to successful extubation(hrs)** |
| --- | --- | --- | --- | --- | --- | --- | --- | --- | --- |
| 16 | 31 | 1/31 | 0/31 | 1/31 | 0/31 | 0.4(0.35-0.6) | 27/31 | 6/31 | 86.4 |
| 17 | 28 | 3/28 | 1/28 | 2/28 | 2/28 | 0.35(0.3-0.51) | 23/28 | 3/28 | 72 |
| 18 | 24 | 3/24 | 3/24 | 0/24 | 0/24 | 0.4(0.29-0.5) | 1/24 | 7/24 | 100.8 |
| 19 | 49 | 9/49 | 1/49 | 8/49 | 5/49 | 0.35 (0.3-0.4) | 47/49 | 8/49 | 74.4 |
| 20 | 5 | 0/5 | 0/5 | 0/5 | 0/5 | 0.35(0.3-0.4) | 5/5 | 0/5 | 225.6 |
| 21 | 33 | 10/33 | 8/33 | 2/33 | 1/33 | 0.3(0.25-0.4) | 5/33 | 8/33 | 86.4 |
| 22 | 52 | 2/52 | 1/52 | 1/52 | 2/52 | 0.35(0.28-0.46) | 18/52 | 11/52 | 86.4 |
| 23 | 82 | 7/82 | 0/83 | 7/83 | 2/82 | 0.4(0.35-0.5) | 69/83 | 20/83 | 64.8 |
| 24 | 2 | 0/2 | 0/2 | 0/2 | 0/2 | 0.88(0.84-0.92) | 2/2 | 2/2 | 182.4 |
| 25 | 45 | 9/45 | 7/45 | 2/45 | 4/45 | 0.4(0.35-0.5) | 5/45 | 3/45 | 86.4 |
| 26 | 24 | 4/24 | 4/24 | 0/24 | 0/24 | 0.35(0.3-0.4) | 6/24 | 3/24 | 57.6 |
| 27 | 47 | 5/47 | 4/47 | 1/47 | 1/47 | 0.38(0.3-0.4) | 14/47 | 12/47 | 100.8 |
| 28 | 58 | 2/58 | 2/58 | 0/58 | 1/58 | 0.38(0.3-0.5) | 2/58 | 9/58 | 98.4 |
| 29 | 75 | 10/75 | 9/75 | 1/75 | 4/75 | 0.4(0.3-0.5) | 4/75 | 12/75 | 62.4 |
| 30 | 59 | 10/59 | 1/59 | 9/59 | 2/59 | 0.4(0.3-0.4) | 55/59 | 7/59 | 76.8 |
| 31 | 70 | 3/70 | 0/70 | 3/70 | 2/70 | 0.39 (0.3-0.39) | 54/70 | 16/70 | 105.6 |
| 32 | 56 | 22/56 | 21/56 | 1/56 | 2/56 | 0.35(0.26-0.4) | 5/56 | 8/56 | 72 |
| 33 | 44 | 12/44 | 1/44 | 11/44 | 2/44 | 0.37(0.3-0.4) | 41/44 | 10/44 | 60 |

**Table S6 - the distribution of variables and median time to successful extubation by site of study.**

| **Variable** | **Coefficient (expressed as exponential of coefficient as log-linked model)** | **95%CI** | **P value** |
| --- | --- | --- | --- |
| Exposure to intervention | 0.88 | 0.75,1 | **0.046** |
| Oxygen Saturation Index | 1.07 | 1.05,1.09 | **<0.001** |
| Corrected gestational age (weeks) | 0.997 | 0.994,1 | 0.1 |
| Term gestation |  |  |  |
| Prematurity 32-37 weeks | 1.09 | 0.97,1.2 | 0.12 |
| Prematurity <32 weeks | 1.57 | 1.37,1.69 | **<0.001** |

**Table S7– Generalised linear model:** Gamma distribution with a log link as duration of IMV is left skewed) demonstrating associations between exposure to the SANDWICH intervention and time to successful extubation following adjustment for known confounders with OSI replacing FiO_2_ to explore impact of inclusion of pressure within the model.
